# X chromosome inactivation across primary human tissues is mostly complete, with significant implications for genetic and clinical studies

**DOI:** 10.1186/s12864-025-12352-9

**Published:** 2025-11-24

**Authors:** Daniel Shriner, Ayo P. Doumatey, Lin Lei, Mateus H. Gouveia, Karlijn A. C. Meeks, Elisabeth F. Heuston, Jie Zhou, Adebowale A. Adeyemo, Charles N. Rotimi

**Affiliations:** 1 Center for Research on Genomics and Global Health, National Human Genome Research Institute, Bethesda, MD USA; 2 Department of Public Health Education, Morehouse School of Medicine, Atlanta, GA USA; 3 Division of Endocrinology, Diabetes, and Nutrition, University of Maryland School of Medicine, Baltimore, MD USA

**Keywords:** Chromosome X, Dosage compensation, Escape, Gene expression, Inactivation

## Abstract

**Background:**

X chromosome inactivation (XCI) refers to silencing of genes on one copy of the X chromosome in XX females, resulting in dosage compensation between XX females and XY males. Genes can escape this silencing, potentially leading to sex-based differences in disease.

**Results:**

Across three primary tissue types, we determined XCI status by integrating whole-genome sequencing with bulk RNA-Seq data to assess allele-specific expression (ASE) at heterozygous SNPs. Across all genes and tissues, the average percentage of individuals showing escape was 4.7%. We show that models of full dosage compensation and no dosage compensation are strongly correlated over most parameter space. For G6PD deficiency, we illustrate that in G6PD*B/G6PD*A- heterozygotes, even if silencing is complete and escape is not associated with disease risk, the allele that is expressed can affect mRNA abundance.

**Conclusions:**

With respect to studies of the genetic architecture of complex traits, our results suggest that a model of full dosage compensation, although not strictly correct for much of chromosome X, is more appropriate than a model of no dosage compensation. We conclude that uncertainty about the model of dosage compensation should not be an impediment to analysis of chromosome X in genetic epidemiology studies.

**Supplementary Information:**

The online version contains supplementary material available at 10.1186/s12864-025-12352-9.

## Background

In mammals, dosage compensation between XX females and XY males is achieved by silencing gene expression in females on one copy of the non-pseudoautosomal region of chromosome X via a process known as X chromosome inactivation (XCI) [1]. Genes that escape XCI show expression from both copies and may contribute to disease and sexual dimorphism [2]. As more single cell types, tissue types, individuals, populations, and species have been examined, evidence is accumulating for a growing number of genes at least partially escaping XCI and for the importance of escape from XCI in phenotypic variation [3–15].

Skewing of XCI refers to a deviation from an equal chance of inactivation of each parental chromosome. Skewing can occur (1) stochastically if XCI is established in an unbalanced way in a limited number of precursor cells or (2) nonrandomly if there is differential proliferation of cells depending on which copy remains active [16]. If skewing is not complete, the resulting cellular mosaicism can confound efforts to identify escape genes.

In genetic epidemiology studies such as association studies, the contribution of chromosome X to phenotypic variation is understudied [17, 18]. Many reasons have been offered, including technical concerns with genotype calling, data cleaning, imputation, and selection of test statistics [19–22]. In particular, several test statistics have been suggested [20, 23–29], but no consensus exists for selecting which test statistic to use.

Here, we integrated bulk RNA and whole genome DNA sequence data to examine XCI in three primary tissue types, subcutaneous adipose tissue, peripheral blood, and skeletal muscle, in 64 females. We assessed gene expression at heterozygous SNPs via allele-specific RNA read counts. We used these data first to estimate sample skewing and then to estimate gene-specific escape given sample skewing. We used these empirical data to inform guidance on accounting for dosage compensation in genetic epidemiology studies. We also examined two genes in more depth, *DUSP9*, which is associated with type 2 diabetes [30–33], and *G6PD*, which is associated with type 2 diabetes [33] and is causal for G6PD deficiency [34].

## Methods

### Samples

Participants were enrolled in Nigeria as part of the Africa America Diabetes Mellitus (AADM) Study, a genetic epidemiology study of type 2 diabetes and related chronic disorders [35]. The case definition of T2D was based on the criteria of the American Diabetes Association (ADA). The criteria were a fasting plasma glucose concentration ≥ 7.0 mmol/L (126 mg/dl) or an oral glucose tolerance test (OGTT) 2 h post-glucose load ≥ 11.1 mmol/L (200 mg/dl) on more than one occasion. A diagnosis of T2D was also accepted if a patient was on physician-prescribed pharmacological treatment for T2D and a review of clinical records showed that pre-treatment fasting glucose and/or OGTT criteria were consistent with the diagnosis. Controls were required to meet the following criteria: fasting plasma glucose < 6.1 mmol/L (110 mg/dl) or OGTT 2 h post-glucose load < 7.8 mmol/L (140 mg/dl) and must have none of the classical symptoms of diabetes (polyuria, polydipsia, and unexplained weight loss). This substudy involved 33 controls and 31 cases.

Baseline blood samples were collected at the time of the main study (exams occurred from 2015 to 2016). Participants were invited to return for a biopsy in which samples were obtained from a single percutaneous needle biopsy of the vastus lateralis muscle (exams occurred from 2016 to 2022). Biopsy samples were immediately dissected into adipose tissue and skeletal muscle tissue, placed in Allprotect Tissue Reagent (www.qiagen.com) to stabilize RNA, DNA, and proteins, and then frozen at −80 °C until extraction for RNA-Seq analysis. At the biopsy visit, a blood sample was collected. Blood samples for RNA-Seq were collected in PAXgene^®^ Blood RNA tubes to stabilize intracellular RNA (www.bdbiosciences.com).

### Nucleic acid extraction, quantification, and quality control

DNA was extracted from buffy coat samples using the PUREGENE^®^ DNA kit (www.qiagen.com) following the manufacturer’s instructions, as previously described [36]. Residual red blood cells were removed by lysis to enrich for leukocytes. Leukocytes were then lysed with an anionic detergent in the presence of a DNA stabilizer. RNA and proteins were removed using RNase A and salt precipitation, respectively. Finally, genomic DNA was recovered by precipitation with alcohol and dissolved in hydration solution (1 mM EDTA, 10 mM Tris-HCl, pH 7.5). DNA samples were quantified by Nanodrop or Qubit (www.thermofisher.com). DNA quality and integrity were assessed by gel electrophoresis before sequencing.

RNA was extracted from blood samples using the E.Z.N.A.^®^ Blood RNA kit (www.omegabiotek.com) following the manufacturer’s instructions. Briefly, cells were collected by centrifugation. Cells were washed and lysed under an optimized buffer containing Proteinase K. Samples were transferred to a Homogenizer Mini Column to remove cell debris and other particulates. Samples were passed through the HiBind^®^ RNA Mini Column, which binds RNA. Genomic DNA was removed from the column by digestion with DNase I. After washing, purified RNA was eluted with RNase-free water.

For adipose tissue and skeletal muscle, RNA was extracted using the E.Z.N.A.^®^ Total RNA Kit II (www.omegabiotek.com). Tissues were first homogenized with RNA-Solv^®^ reagent that inactivates RNases. After adding chloroform, a homogenate was separated into aqueous and organic phases by centrifugation. The aqueous phase, which contains RNA, was passed through a HiBind^®^ RNA Mini column to extract RNA. After washing, RNA was eluted in DEPC water. All RNA samples were quantified using Nanodrop (www.thermofisher.com) and quality was assessed by a TapeStation System (www.agilent.com).

### Whole-Genome sequencing

PCR-free libraries were generated from 1 µg genomic DNA using the TruSeq DNA PCR-Free HT Sample Preparation Kit (Illumina). Median insert sizes were approximately 400 bp. Sequencing was performed to generate 151 bp paired-end reads at 30× read depth using the Illumina NovaSeq 6000 platform at the NIH Intramural Sequencing Center (NISC). Reads were aligned to the GRCh38 (hg38) decoy reference genome, including alternate contigs, using the Burrows-Wheeler Aligner [37]. Using PICARD version 2.27.3, proper alignment of paired-end reads was ensured using FixMates and duplicate reads were marked with MarkDuplicates [38].

Base Quality Score Recalibration was performed using GATK, using known SNPs and indels from dbSNP138 and Mills-Devine 1000G gold standard sites for model training [39–41]. Variant calling was conducted using GATK HaplotypeCaller, with ploidy set to 2 for both the pseudoautosomal regions (PARs) and non-pseudoautosomal region (non-PAR) of chromosome X. GVCFs were consolidated using GATK GenomicsDB, followed by joint genotyping. Variant Quality Score Recalibration was applied to SNPs using 1000G Omni2.5 and HapMap 3 variants as training sets. Additional filtering criteria included a maximum missingness threshold of 0.5, minimum quality score (minQ) of 30, and a minor allele count (MAC) ≥ 3. Processing of vcfs was performed using vcftools version 0.1.16 [42]. Phasing was performed using the Eagle version 2.4 algorithm [43] and the r3 reference panel used by the TOPMed Imputation Server [44, 45].

### Whole-Transcriptome sequencing

Libraries were generated from 200 to 1000 ng total RNA using the TruSeq Stranded Total RNA with Ribo-Zero Globin Kit (Illumina). Sequencing was performed to generate a minimum of 50 M 151 bp paired-end reads per library using the Illumina NovaSeq 6000 platform at NISC. Following best practices recommendations [46], we used fastp version 0.23.4 with options -D -p -l 151 -x to clean the fastq files [47]. Reads were aligned to the hg38 reference genome without alternate scaffolds, patches, or chromosome Y using STAR version 2.7.11b in two-pass mode [48, 49]. To reduce reference bias, we used WASP as implemented in STAR [50]. We retained uniquely mapping primary alignments in proper pairs [51].

### Allele-Specific expression

We used the GATK (version 4.6.0.0) tool ASEReadCounter to integrate WGS and RNA-Seq data. We then used the R package XCIR (version 1.6.0) to analyze allele counts [52]. XCIR implements a two-step strategy. The determination of active and inactive X chromosomes differs from cell to cell, leading to cellular mosaicism. Thus, the first step is to estimate, for each sample, how much cellular mosaicism departs from 50:50, referred to as XCI skewing. This step accounts for the possibility that a heterozygous SNP is actually homozygous (i.e., a sequencing error). This step also accounts for the possibility that a gene that is commonly silenced may escape in a given individual or tissue. The second step is to infer the XCI state for each gene that includes an informative (i.e., heterozygous) SNP with sufficient read depth by comparing the allele counts with the sample’s level of XCI skewing. For a given gene, XCIR tests the null hypothesis that the ASE ratio of an escape gene equals the sample’s skewing against the alternative hypothesis that the ASE ratio of an escape gene is greater than the sample’s skewing. Genes with a more balanced ASE ratio than the sample’s skewing are inferred to have escaped XCI. To prevent bias towards genes with high levels of gene expression, we kept SNPs with ≥ 1 read (summed across alleles). To prevent bias towards escape, we kept SNPs with ≥ 0 reads on each allele. To calculate allele-specific expression per gene per individual, we used the most highly expressed SNP (total reads across both alleles) per gene. We used a mixture model to estimate mosaicism and XCI escape, followed by formal hypothesis testing of a null hypothesis of silencing vs. an alternative hypothesis of escape [52]. Across individuals, a gene with a percent escape (PE) = 0% was declared completely silenced, 0% < PE ≤ 25% was declared mostly silenced, 25% < PE < 75% was declared variably escaped, 75% ≤ PE < 100% was declared mostly escaped, and PE = 100% was declared completely escaped.

### Overrepresentation analysis

We used the PANTHER version 19.0 classification system to test for overrepresentation of PANTHER pathways, PANTHER protein classes, PANTHER GO-Slim molecular functions, PANTHER GO-Slim biological processes, PANTHER GO-Slim cellular components, and Reactome pathways [53, 54].

### Genotype coding for genome-wide association studies

Assume that Hardy-Weinberg equilibrium holds (in females) and that allele frequencies do not vary between sexes. (Relaxing each assumption requires one additional parameter.) Let $\:p$ represent the frequency of the reference allele and let $\:q$ represent the frequency of the alternate allele, subject to the constraint $\:p+q=1$. Let $\:M$ represent the number of males and let $\:F$ represent the number of females. For the non-pseudoautosomal region, we use genotype coding (0,1) in males and (0,1,2) in females under no dosage compensation and (0,2) in males and (0,1,2) in females under full dosage compensation [23]. Intuitively, these two models differ only in the number of males with the alternate allele. Under no dosage compensation, the expected genotypic value is

$$\:\frac{M}{M+F}q+\frac{F}{M+F}2q.$$

Under full dosage compensation, the expected genotypic value is

$$\:\frac{M}{M+F}2q+\frac{F}{M+F}\left(2pq+{2q}^{2}\right)=2q.$$

The covariance is

$$\:2pq.$$

Under no dosage compensation, the variance of the genotypic value is

$$\:\frac{M}{M+F}q\left(1-\frac{M}{M+F}q\right)+\frac{F}{M+F}2pq.$$

Under full dosage compensation, the variance of the genotypic value is

$$\:2pq\frac{2M+F}{M+F}.$$

The correlation coefficient $\:r$ between the coding systems is then given by

$$\:r=\frac{2pq}{\sqrt{\frac{M}{M+F}q\left(1-\frac{M}{M+F}q\right)+\frac{F}{M+F}2pq}\sqrt{2pq\left(\frac{2M+F}{M+F}\right)}}.$$

If $\:M=0,\:r=\frac{2pq}{\sqrt{2pq}\sqrt{2pq}}=1$, and if $\:F=0$, $\:r=\frac{2pq}{\sqrt{pq}\sqrt{4pq}}=1$.

## Results

### Genic coverage

After data cleaning, we had DNA and RNA sequencing data from 29 baseline blood samples. We also had DNA and RNA sequencing data from 36 subcutaneous adipose tissue samples, 41 blood samples, and 42 skeletal muscle samples from the time of biopsy. These samples were derived from a total of 64 participants with an average age at baseline of 55.8 years (Additional file 1). Based on DNA, there were 580,745 SNPs on chromosome X in these individuals. There are 844 protein-coding genes on chromosome X in the hg38 human reference gene set. After integrating the RNA-based read counts with heterozygous SNPs, up to 87.4% of protein-coding genes remained for analysis. The remainder of protein-coding genes were not assessed because of a lack of heterozygous SNPs or RNA reads.

### Skewing across tissues

**Fig. 1:**
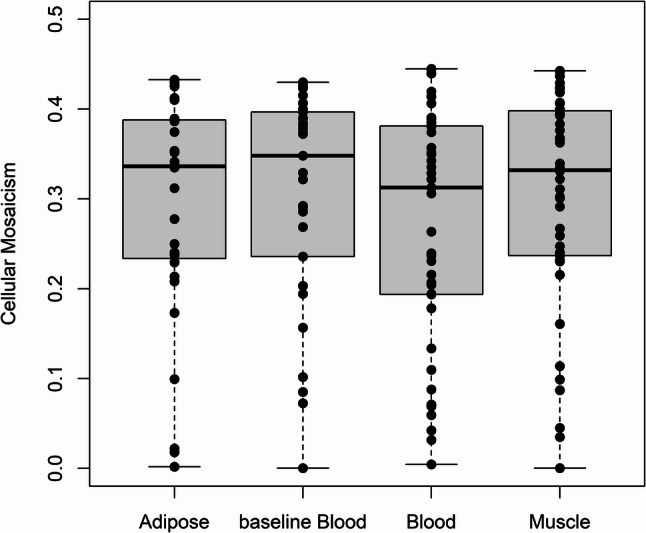
Estimated levels of cellular mosaicism. Each dot corresponds to an individual. The bar represents the median, the box represents the interquartile range, and the whiskers represent the range. A value of 0 indicates that a given allele is actively expressed is no cells (complete skewing) and a value of 0.5 indicates that a given allele is actively expressed in half of cells (no skewing)

The level of cellular mosaicism or skewing is a key parameter in inferring escape from XCI. Within tissue, skewing was variable across individuals and closer to no skewing than complete skewing (Fig. 1), consistent with previous findings across 45 adult tissues [55]. Across tissues, the median level of skewing was not different (Kruskal-Wallis rank sum test, *p* = 0.609). These results indicate that some individuals will be more informative than others for inferring escape from XCI but also imply that escape could be underestimated in many individuals.

### Variability in XCI escape across tissues

**Fig. 2:**
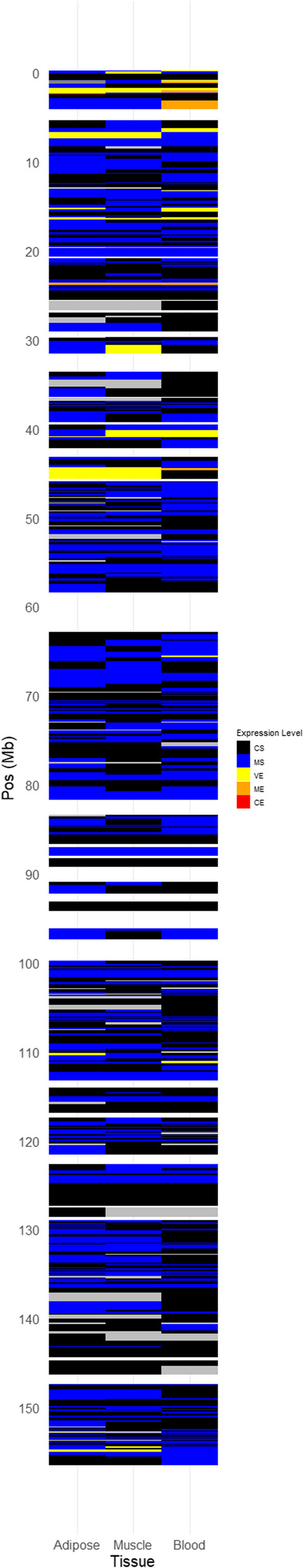
X-linked genes are mostly silenced. Each column corresponds to a tissue and each row corresponds to a gene. White represents genes not expressed in any of the tissues and gray represents genes not expressed in a specific tissue. The X chromosome inactivation status was determined by calculating the percentage of females in whom that gene escaped inactivation. CS refers to completely silenced (black), MS refers to mostly silenced (blue), VE refers to variably escaped (yellow), ME refers to mostly escaped (orange), and CE refers to completely escaped (red). The data for this figure are provided in Additional file 2 and summary counts by tissue and category are provided in Additional file 3

Most X-linked genes showed a low level of escape from XCI (Fig. 2). In adipose tissue, we interrogated 686 genes, covering 81.3% of protein-coding genes. Across genes and individuals, the mean percentage of escape was 4.7%. In peripheral blood, we interrogated 708 genes, covering 83.9% of protein-coding genes. Across genes and individuals, the mean percentage of escape was 5.7%. In skeletal muscle, we interrogated 685 genes, covering 81.2% of protein-coding genes. Across genes and individuals, the mean percentage of escape was 3.9%.

Across all three tissues, we observed gene expression for a total of 727 genes. Of these genes, 68.5% were not completely silenced in all tissues. Across all genes and tissues, the average percentage of individuals showing escape was 4.7%. These results indicate that (1) most genes escape XCI inactivation, (2) escape tends to occur in a small number of individuals, and (3) escape is at least partially tissue-specific. Of the 727 genes, 29 showed a tissue-averaged level of escape ≥ 25%. These 29 genes included 10 that map to PAR1 (*GTPBP6*, *PPP2R3B*, *CSF2RA*, *SLC25A6*, *ASMTL*, *P2RY8*, *AKAP17A*, *DHRSX*, *ZBED1*, and *CD99*), three with nonfunctional homologs on chromosome Y (*GYG2*, *PRKX*, and *TXLNG*), and six with functional homologs on chromosome Y (*EIF1AX*, *ZFX*, *USP9X*, *DDX3X*, *KDM6A*, and *RPS4X*). Among these 29 genes, there was no overrepresentation of pathways, molecular functions, biological processes, cellular components, or protein classes.

### Variability in XCI escape over time

To assess the stability of silencing over time, we compared baseline blood to blood obtained during tissue biopsy. Seven participants for whom we had baseline blood samples returned for the biopsy visit, with an average of 2.3 years between visits. For this analysis, we added 22 participants for whom we had results from the baseline visit but no biopsy data and 34 participants for whom we had results from the biopsy visit but no baseline data, with an average of 2.7 years between visits. In the baseline blood sample, we detected expression of 738 genes, covering 87.4% of protein-coding genes. Across genes and individuals, the mean percentage of escape was 6.5%.

**Fig. 3:**
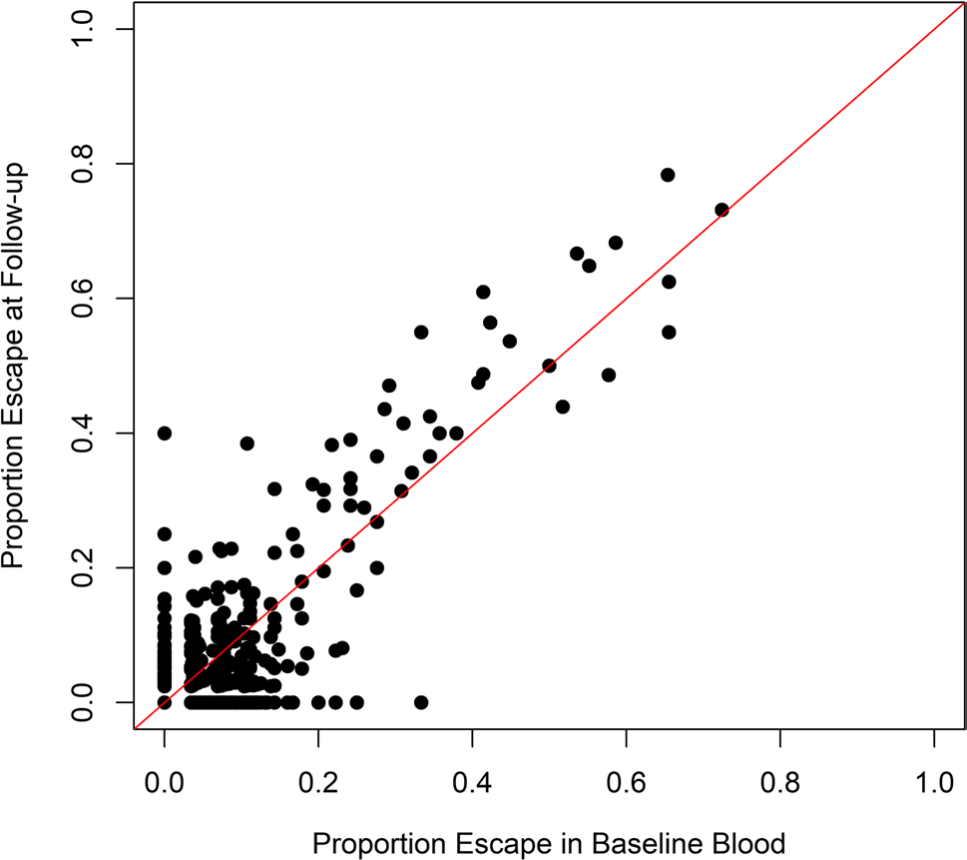
(In)stability of escape over time in peripheral blood

We compared the level of skewing between baseline blood and blood obtained during tissue biopsy (Fig. 1). Between baseline and follow-up, the median level of skewing was not different (Kruskal-Wallis rank sum test, *p* = 0.260). We detected gene expression of 706 genes in common in peripheral blood between the visits. The percentage escape between the two visits was positively correlated ($\:r=0.794$), with 23.8% of genes showing more silencing at the later visit and 13.7% of genes showing more escape at the later visit (Fig. 3). Genes that constitutively escape XCI generally avoid inactivation during development and are predominantly genes with homologs on chromosome Y [56]. Genes that facultatively escape XCI are inactivated during development but are later reactivated, and the characteristics of genes that facultatively escape XCI are unclear [56]. To gain insight into the three patterns of gene expression, we performed gene set overrepresentation analysis. The gene set with a stable pattern of gene expression was enriched for negative regulation of transcription by RNA polymerase II ($\:p=1.94\times\:{10}^{-10}$), nucleus ($\:p=2.27\times\:{10}^{-4}$), extracellular space ($\:p=2.50\times\:{10}^{-5}$), chromatin/chromatin-binding or regulatory protein ($\:p=6.41\times\:{10}^{-4}$), scaffold/adaptor protein ($\:p=1.58\times\:{10}^{-4}$), and defense/immunity protein ($\:p=1.03\times\:{10}^{-4}$). The gene set that showed increased escape was enriched for replisome ($\:p=6.68\times\:{10}^{-4}$) and mediator complex ($\:p=6.33\times\:{10}^{-4}$). The gene set that showed increased silencing was enriched for regulation of cell migration ($\:p=3.80\times\:{10}^{-5})$ and transcription cofactor ($\:p=3.00\times\:{10}^{-6}$).

### Genotypic coding for dosage compensation

**Fig. 4:**
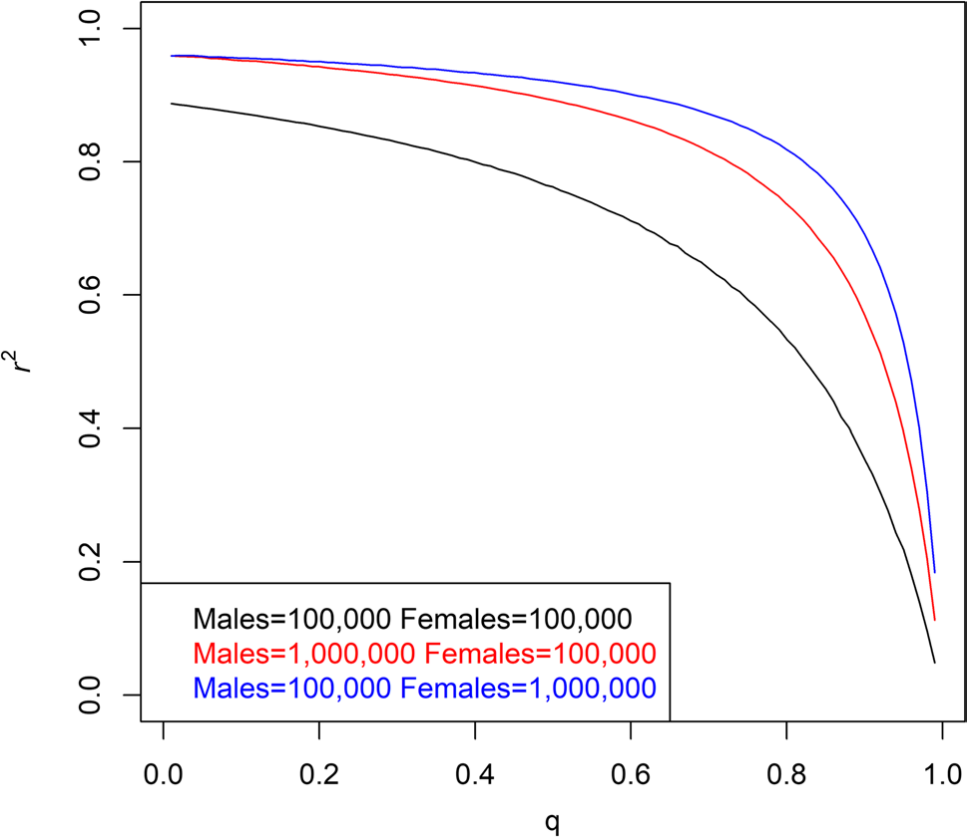
Correlation between full and no dosage compensation. The frequency of the effect allele is given by $\:q$. If $\:q<0.5$, then the effect allele is also the minor allele

In association studies, testing genetic variants on the non-pseudoautosomal region of chromosome X requires a model of genotypic coding that accounts for dosage compensation. The model of full dosage compensation assumes that males with one copy of the effect allele are equivalent to females with two copies of the effect allele whereas the model of no dosage compensation assumes that males with one copy of the effect allele are equivalent to females with one copy of the effect allele [23]. To explore the consequences of choosing between these two models, we determined the correlation between the two genotypic coding schemes. When the effect allele is also the minor allele, the correlation between genotypic coding under full and no dosage compensation is at least 0.762, indicating strong correlation between the two schemes (Fig. 4). Given our finding that the average percentage of individuals showing escape was 4.7% across all genes and tissues, the full dosage compensation model is more appropriate than the no dosage compensation model. Correlation is weakest when the numbers of males and females are equal (Fig. 4), and dosage compensation is not an issue for a sex-specific study.

### Disease implications

Given that the parent study was a genetic epidemiology study of type 2 diabetes, we explored the implications of XCI for *DUSP9*, the chromosome X gene most strongly associated with type 2 diabetes [30–33]. *DUSP9* showed little to no gene expression in the three tissues we examined, consistent with GTEx data. We next explored the implications of XCI at *G6PD*, another chromosome X gene associated with type 2 diabetes [33, 57]. *G6PD* was completely silenced in both adipose tissue (31/31 individuals) and skeletal muscle (36/36 individuals) and mostly silenced (34/36 individuals) in peripheral blood. Escape from XCI was not associated with case/control status ($\:p=0.992$).

Mutations in *G6PD* cause glucose-6-phosphate-dehydrogenase deficiency, which can lead to hemolytic anemia in the presence of oxidative stress. G6PD*B is the wild type, with normal enzyme activity. The presence of rs1050829 defines the G6PD*A variant, which has enzyme activity in the normal range [58]. If rs1050828 is also present, enzyme deficiency can result and the variant is known as G6PD*A- [59]. Using the ASE results, we identified 20 individuals in whom *G6PD* was silenced in peripheral blood and for whom rs1050829 was the most expressed SNP in *G6PD*. Next, using the phased WGS data, we stratified those individuals as G6PD*A (rs1050829) or G6PD*A- (rs1050829 and rs1050828). Then, we compared total read counts as a function of which haplotype was silenced. In G6PD*B/G6PD*A individuals, gene expression was similar regardless of which haplotype was silenced (Table 1). In contrast, in G6PD*B/G6PD*A- individuals, gene expression trended lower if the G6PD*A- haplotype was expressed (Table 1). These results provide evidence that which allele is expressed can matter, even in the absence of escape.

**Table 1:** Gene expression at rs1050829 as a function of XCI

G6PD Diplotype	Haplotype Silenced	Mean Total Read Count
B/A	B	69.7
	A	79.6
B/A-	B	42.7
	A-	106.1

## Discussion

Initial studies of XCI were performed in a single cell type, such as fibroblasts or lymphoblastoid cell lines. Subsequent meta-analysis suggested that 51% of X-linked genes are completely silenced and 8% had completely escaped [8]. Here, we investigated XCI in three different primary tissue types. In any one tissue type, we found that from 48.2% to 55.8% of genes were completely silenced and 0% had completely escaped. Compared to a previous estimate that incomplete XCI affects at least 23% of X-linked genes [12], we found that incomplete XCI affects 68.5% of assayed X-linked genes. A partial explanation for the large difference between these estimates is that one criterion used previously to declare XCI status was biased against escape: genes classified as silenced under one condition and classified as variably escaped in another condition were declared silenced.

The determination of which X chromosome is inactivated varies from cell to cell. Consequently, analysis of bulk RNA-seq data must account for cellular mosaicism or skewing. The method we used to identify escape genes depends on skewing but does not require complete skewing. By testing a gene’s ASE ratio compared to the sample’s skewing, the method has the most power to detect escape if skewing is complete and has no power if there is no skewing. Among the individuals and tissues we examined, the amount of skewing tended to be small. Therefore, it is possible that the level of escape was underestimated.

Genes that escape XCI tend to be characterized by the existence of a homolog on the Y chromosome [2]. For example, genes within the PARs tend to escape XCI. Escape from XCI could reflect failure of silencing (constitutive escape) or reactivation after silencing (facultative escape). Our study was not designed to distinguish between these two possibilities, but it is tempting to hypothesize that a general pattern of escape in a small number of individuals for any given gene is more likely to reflect facultative escape than constitutive escape.

Comparison of blood samples from two time points suggested that patterns of mosaicism are stable over time whereas patterns of silencing and escape varied over time. Changes in gene expression levels in adults may reflect environmental exposures that affect methylation. Most, but not all, genes have the same XCI status predicted by gene expression or by methylation across tissues; however, methylation patterns differ across tissues for a minority of genes, leading to differences in XCI status inferred from gene expression or from methylation [5]. Another issue is that the blood composition changes over time. Overrepresentation of genes annotated with regulation of cell migration in the subset of genes that showed increased silencing could reflect resolution of infection. Each of the three tissue types we examined are composed of multiple cell types. Therefore, variation in XCI over time may reflect tissue heterogeneity, rather than changes in XCI status. To address this issue, we support the study of XCI at single cell resolution [60].

One strength of our study is the investigation of primary samples from multiple tissues, as opposed to cell lines. Skeletal muscle was sampled for investigation of glucose utilization in type 2 diabetes and adipose tissue was sampled for investigation of lean vs. obese cases of type 2 diabetes. Another strength is the use of RNA-Seq instead of expression arrays. Expression arrays are susceptible to confounding by technical covariates because read counts are compared between samples, whereas allele-specific expression analysis is based on comparison of read counts within samples [46].

One limitation of our study is that analysis of allele-specific expression requires heterozygous SNPs. This requirement affects the analysis in two ways. One, there must be at least one heterozygous SNP in a gene in an individual or the gene will be invisible to the analysis. Two, the power of the analysis will be limited by the number of individuals with a heterozygous SNP in a gene. A second limitation is that gene expression levels might be too low to be detected by bulk tissue RNA-Seq. It has been suggested that analysis of only highly expressed genes may enrich for dosage-sensitive genes [61].

The method we use to infer escape from XCI depends on estimation of skewing in the sample. When skewing is low, the hypothesis test is underpowered to detect escape. If gene expression levels are low, reads may stochastically come from only one chromosome, giving the appearance of silencing. Given these biases towards inference of silencing, our finding of a low level of escape that varies by gene, tissue, and individual does not seem to be an artifact of the method.

We explored the implications of XCI with respect to *DUSP9* and *G6PD* in the context of type 2 diabetes. We were not able to regress case-control status on XCI status for *DUSP9* because the gene was not expressed in adipose tissue, peripheral blood, or skeletal muscle. This finding is not surprising, as GTEx data showed that gene expression of *DUSP9* is restricted to the kidneys. Also, we were not able to regress case-control status on XCI status for *G6PD* in adipose tissue and skeletal muscle because the gene was completely silenced in both tissues. In peripheral blood, *G6PD* was mostly silenced, and both individuals in whom *G6PD* escaped XCI were type 2 diabetes controls.

In the context of G6PD deficiency, we found that in G6PD*B/G6PD*A heterozygotes, gene expression did not appear to vary according to which allele was silenced. In contrast, in G6PD*B/G6PD*A- heterozygotes, gene expression trended lower if G6PD*B was silenced than if G6PD*A- was silenced. These results are consistent with a contribution of XCI to the clinical presentation of G6PD deficiency [62], and these results warrants further investigation in a larger sample. It should be noted that hemolytic anemia involves mature erythrocytes, not peripheral blood mononuclear cells, so ASE in peripheral blood should not be overinterpreted.

Our results should facilitate integration of chromosome X into studies of the genetic architecture of complex traits [17, 18, 20]. First, if the effect allele is the minor allele, then genotypic coding schemes based on full vs. no dosage compensation are strongly positively correlated, regardless of the numbers of males and females, explaining why statistical power between the two schemes is so similar [27]. Second, genotypic coding can vary by gene in tissue-specific ways and tissues contain multiple cell types, so association analysis along chromosome X should be performed separately by cell type. However, in practice, at the whole organism level at which association studies are performed, genotypic coding is driven by the level of XCI averaged across all tissue or cell types. Given that we observed 95.3% silencing averaged over genes, tissues, and individuals, genotypic coding based on the full dosage compensation model is appropriate for most of chromosome X [28, 63], at least as a first pass analysis, and little power will be lost if the full dosage compensation model is used for genes that have escaped XCI. Alternatively, a test that maximizes over different patterns of XCI could be used [29], with a loss of power resulting from a correction for multiple testing.

## Conclusions

Our results suggest that silencing is variable but mostly complete across genes, tissues, and individuals. In any one tissue type, we found that approximately half of genes were completely silenced in all individuals. Across three tissue types, we found that 31.5% of genes were completely silenced in all individuals. Our results support the hypothesis that the number of variably escaped genes will increase as more primary tissue types are examined [14]. Similar to early work in lymphoblastoid cell lines [64], our findings suggest that dosage compensation is close to complete, and that the potential contribution of escape from XCI to phenotypic differences between the sexes may be limited.

## Supplementary Information

Supplementary Material 1.

Supplementary Material 2.

Supplementary Material 3.

## Data Availability

The WGS and RNA-Seq data are available through dbGaP accession number phs001844.v2.p1 (https://www.ncbi.nlm.nih.gov/projects/gap/cgi-bin/study.cgi? study_id=phs001844.v2.p1). Custom code used in this paper is available through GitHub (https://github.com/dshriner/XCI/tree/main).
